# Untargeted Metabolomics Reveals the Effect of Selective Breeding on the Quality of Chicken Meat

**DOI:** 10.3390/metabo12050367

**Published:** 2022-04-19

**Authors:** Kai Shi, Qian Zhao, Minghui Shao, Ying Duan, Dongfeng Li, Yangqing Lu, Yanfei Tang, Chungang Feng

**Affiliations:** 1 College of Animal Science and Technology, Nanjing Agricultural University, Nanjing 210095, China; 2020205003@stu.njau.edu.cn (K.S.); zhaoqian0117@gmail.com (Q.Z.); 2019105007@stu.njau.edu.cn (M.S.); 2020105006@stu.njau.edu.cn (Y.D.); lidongfeng@njau.edu.cn (D.L.); 2 State Key Laboratory for Conservation and Utilization of Subtropical Agro-Bioresources, College of Animal Science and Technology, Guangxi University, Nanning 530000, China; lyq@gxu.edu.cn; 3 Guangxi Fufeng Agricultural and Animal Husbandry Group Co., Ltd., Nanning 530000, China; 18853857810@163.com

**Keywords:** metabolomics, selection, meat quality, skeletal muscle

## Abstract

The selection for improved body weight is an effective approach in animal breeding. Guangxi Partridge chickens have differentiated into two lines under selective breeding, which include line S and line D that have shown statistically significant differences in body weight. However, the meat quality analysis in our study indicated that the quality of breast and thigh muscles in line S chickens changed, which included increased values of L*, b*, and drip loss and decreased a* value, pH, and shear force in skeletal muscles. To illuminate the effect of selection on skeletal muscles, LC-MS/MS metabolomics was performed to explore differentiated metabolites in divergent tissues from the two chicken lines. The results of principal component analysis and orthogonal projection to latent structures discriminant analysis suggested that metabolites of different groups were separated, which suggested that selective breeding certainly affected metabolism of skeletal muscles. KEGG analysis identified that valine, leucine, and isoleucine biosynthesis, glycerophospholipid metabolism, and glutathione metabolism noteworthily changed in breast muscle. Amino sugars and nucleotide sugar metabolism, ascorbate and aldarate metabolism, the pentose phosphate pathway, pentose and glucuronate interconversions, fructose and mannose metabolism, and glycerophospholipid metabolism were remarkedly identified in thigh muscle. These screened pathways suggested oxidative stress in breast and thigh muscles, which corresponded with our previous results. Therefore, this study determined that glycerophospholipid metabolism conservatively functioned in muscle flavor and development but exhibited different anti-oxidative patterns in different skeletal muscles. Overall, the present study identified several differentiated metabolites and pathways for exploring differences in meat quality between different broiler populations.

## 1. Introduction

The direction of animal domestication depends on the necessities of humans, which results in the variable traits of domesticated animals. These alterations are not only restricted to qualitative characteristics, such as colors of skin or morphologies of combs in broiler chickens, they have also contributed to changes in quantitative characters that include most economic traits of livestock [1,2,3]. Among them, elevated yield and quality of meat are important breeding targets. For example, myostatin is a negative regulator in muscle development, and animals with the myostatin mutation produce more muscle mass [4]. Currently, an effective breeding method is selecting myostatin-mutant individuals to propagate high-performance offspring in Bos taurus and Coturnix japonica [4,5,6]. During the selection of 11 generations for AFP (abdominal fat percentage), the fat chicken line and lean chicken line were produced from the same ancestors, and Wang et al. found that the level of AFP was 5.29 times higher in the fat chicken line than in the lean one [7]. Peking duck, which is a famous breed that comprises 70% of the market share of duck consumption, has differentiated into lean and fat populations for braising and roasting, respectively, to satisfy dietary requirements [8]. Therefore, selection exerts significant effects on animal performances.

However, high performance in meat production is easily accompanied by changes in meat quality, which is generally reflected by meat color, pH, water holding capacity, tenderness, and flavor. In recent decades, turkeys have been selected for fast growth, which has resulted in distinct muscular abnormalities, such as white strings in the ventral area of breast muscle, although this seemed to have no influence on the birds’ health [9]. Meat pH showed higher levels in broilers after selection for 13 generations, and meat redness (a*), yellowness (b*), and meat drip loss decreased notably [10]. Pale, soft, and exudative (PSE) meat was first found in selected Sus scrofa; inferior meat was caused by the mutation of the ryanodine receptor (RYR1) [11]. PSE was also displayed in fast-growth fowl, but the mechanism was unclear. It is generally thought that selective pressure increases sensitivity of broilers and changes the binding activity of calcium channel ryanodine in skeletal muscle [12]. Aerobic and anaerobic metabolism result in differentiated metabolites in skeletal muscle that determine meat quality. Skeletal muscle is composed of oxidative, oxidative glycolytic, and glycolytic myofibers, which process unique metabolic patterns and physiological functions [13]. Rapid growth induced abnormal muscle structure, large myofiber diameters, increased the number of glycolytic myofibers, and induced low proteolytic ability [14]. Huo et al. compared glycolytic potential between different chicken breeds and found that chickens with fast growth possessed high glycolytic potential in breast and thigh muscles, which indicated that the metabolic pattern was converted under different growth rates [15]. Skeletal muscle with a high proportion of glycolytic type myofibers was denser, lighter, and more transudative, which resulted in inferior meat quality [16]. These results indicated that rapid growth transformed metabolic patterns in skeletal muscle and contributed further to changed meat quality.

Metabolomics is a novel technique to detect the alteration of endogenic small metabolites that are affected by outer stimulation or inner disruption, and it is a way to diagnose and predict the mechanism of alterations [17,18]. There are several detection methods in metabolomics, such as GS-MS (gas chromatography coupled with mass spectrometry), NMR (nuclear magnetic resonance), LC-MS (liquid chromatography coupled with mass spectrometry), and LC-MS/MS. LC-MS possesses wide coverage of substances, and it is considered the most stable and suitable technique to study metabolism. LC-MS/MS is based on LC-MS, reducing background noise to acquire more refined results, and has been widely employed for scientific research [19]. Li et al. used LC-MS/MS to explore the transformation of metabolites during oocyte development, and they found that the concentration of arachidonic acid (ARA) decreased in the meiotic maturation stage. Subsequently, rescue experiments determined that ARA was an inhibitor of oocyte ripening [20]. LC-MS/MS also has been performed to screen for differentiated metabolites during myogenesis. Pantothenate, a coenzyme, and arginine played dominant roles in the proliferation stage, but biosynthesis of vitamin B6, glyoxylate, and nitrogen were important during differentiation; these results provided theoretical support for muscle development and pathology [21]. Xiao et al. compared native and commercial chicken breeds by GS-MS and found that the contents of water-soluble molecular weight compounds and fatty acids were significantly higher in muscle from native breeds [22]. Xiao et al. also used the metabolome to analyze the differentiated metabolites between different periods in Wuding chicken, and identified that several substances were changed in time-dependent mode, which provided the suggestion of a suitable slaughter time for producers [23]. These results contribute to better understanding the difference of meat quality in different chicken populations or muscle development.

Until now, previous studies have focused mainly on clarifying the mechanism of differentiated growth performance and meat quality among disparate chicken breeds, but they rarely focused on the impact of selection on meat quality. Guangxi Partridge chicken is a native breed in China, and breeding lines S and D have been selected for four generations for high performance [24]. Line S has been under selection for higher growth rate, while line D has been selected for egg production. This breed is usually reared for 90–130 days to reach market weight. At the age of 90 days, the body weight of line S cockerels is about 1.7 kg, and the body weight of line D cockerels is about 1.5 kg after selective breeding [24]. Therefore, these lines represent a perfect model to explore the effect of artificial selection on meat quality. In the present study, these two lines of chickens were raised for 90 d and were subsequently harvested to obtain breast and thigh muscles. After physical properties were analyzed, LC-MS/MS was adopted subsequently to determine variation in metabolites in skeletal muscle of the two populations.

## 2. Results

### 2.1. Quality Parameters of Breast and Thigh Muscles from Two Chicken Lines

**Figure 1:**
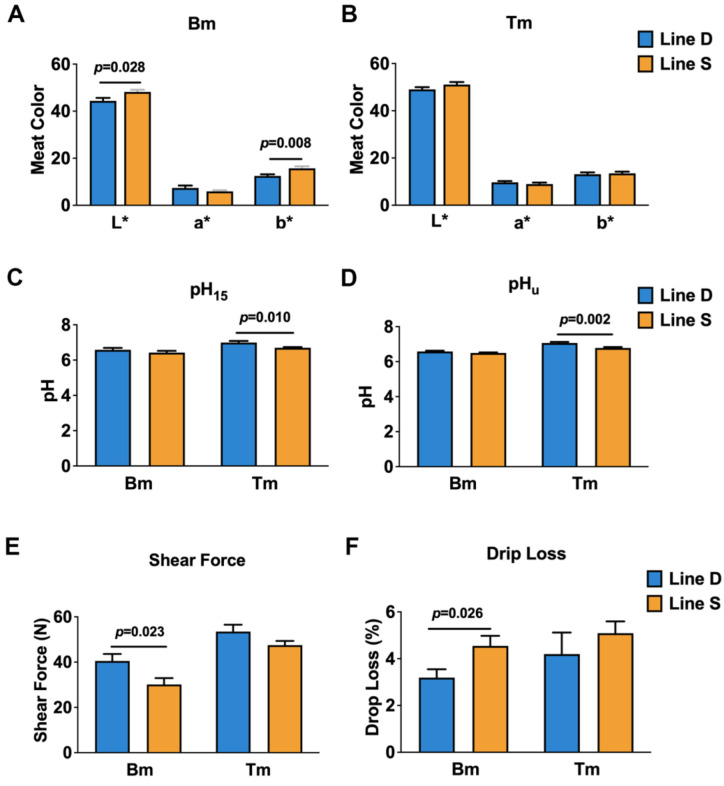
Comparison on quality characteristics of breast and thigh muscles from line S and line D Guangxi Partridge chickens. (**A**,**B**) indicate meat color in breast and thigh muscles, respectively, between line S and line D chickens, where L* represents lightness, a* represents redness, and b* represents yellowness. (**C**,**D**) show the pH of breast and thigh muscles from different lines, respectively. pH_15_ indicates the pH measured at 15 min after slaughter, and pH_u_ indicates the pH at 24 h after slaughter. (**E**,**F**) show the level of shear force and drip loss of two muscles from different lines. Bm, breast muscle; Tm, thigh muscle. Values are shown as mean ± SD (*n* = 10 individuals per group).

Quality characteristics of skeletal muscle from two groups were detected to explore the effect of selection pressure on meat quality, which included meat color (L* = lightness, a* = redness, and b* = yellowness), pH, shear force, and drip loss (Figure 1). Values of L* and b* were improved in breast and thigh muscles from line S chickens; however, there was only statistically significant difference in L* and b* in breast muscle. In addition, contrasted with line D chickens, a* values between breast and thigh muscles in line S decreased slightly (Figure 1A,B). The level of pH_15_ was decreased in breast and thigh muscles from line S cocks at slaughter, and there was a considerable change in the thigh muscle (Figure 1C). Twenty four hours after slaughter, pH_u_ also declined slightly in breast; however, pH_u_ improved in thigh muscle from the line S group (Figure 1D). Shear force of different muscles reduced in selected birds, although breast muscle showed a greater decline (*p* < 0.05) (Figure 1E). Drip loss in the line S group was notably higher in breast muscle, but there was a slight improvement in thigh muscle (Figure 1F).

### 2.2. Quality Control of Metabolomics from Muscle Samples

The metabolomics analysis based on the LC-MS/MS detection technology was adopted to reveal metabolite differences in skeletal muscles from two chicken lines. To acquire reliable data for metabolomic detection, QC (quality control) samples were utilized generally [25]. In this study, four pooled QC samples were obtained by mixing equal aliquots of all the samples, and the values for Pearson correlation coefficient were 1.00, which indicated that this method was stable and repeatable. The identified metabolites from breast muscle separated from thigh muscle (Figure S1). In addition, one thigh muscle from line S and one thigh muscle from line D had a lower correlation with the other thigh tissue samples and were not used for further analysis (Figure S1).

### 2.3. Multivariate Statistical Analysis of Metabolomics

**Figure 2:**
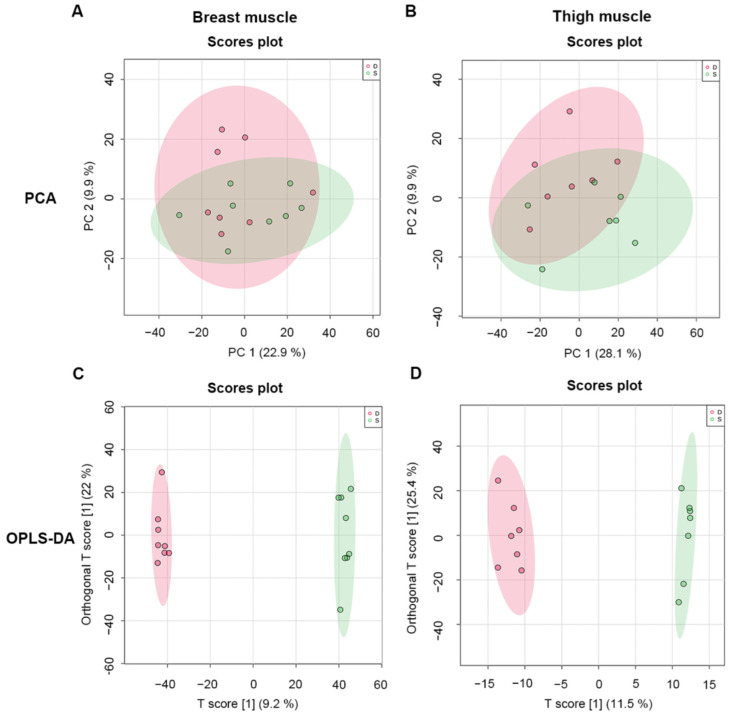
Multivariate analysis of metabolomics data for skeletal muscle from different groups. (**A**,**B**), PCA graphs of breast and thigh muscles between line S and line D chickens, respectively. (**C**,**D**), OPLS-DA score plots of metabolomic assay of breast and thigh muscles of chickens from different lines, respectively. D, line D; S, line S.

A total of 1332 metabolites were screened from breast and thigh muscles under positive and negative ion modes. To assess the differences in metabolites from intergroup samples, PCA and OPLS-DA were used. The PCA of metabolites of breast and thigh muscles from line S chickens were separated from line D broilers, which indicated that metabolites of skeletal muscles changed under selective breeding (Figure 2A,B). Subsequently, the OPLS-DA model was used to distinguish differentiated metabolites that were obtained from screened metabolic profiles, in which the sample cluster from line S was divergent from line D (Figure 2C,D).

### 2.4. Screening and Correlation Analysis of Differentiated Metabolites

**Figure 3:**
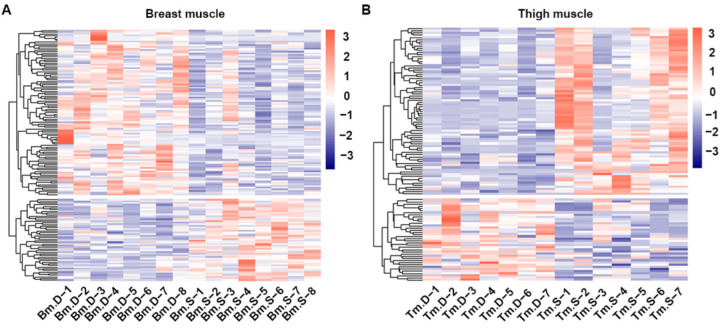
Analysis of differentiated metabolites in skeletal muscles from two chicken lines. Heatmap of hierarchical clustering of differentiated metabolites in breast (**A**) and thigh (**B**) muscles between line D and line S. Bm.D and Tm.D in the plot indicate samples from breast and thigh muscles from line D; Bm.S and Tm.S indicate breast and thigh muscles from line S.

The screening criteria used to select differentiated substances in the breast and thigh muscles of broilers from line S and line D were VIP > 1 and *p* value < 0.05. We identified 151 and 115 differentiated metabolites in the breast and thigh muscles, respectively, by positive and negative ion modes (Tables S1 and S2). Fifty metabolites had a higher concentration, and 101 had a lower concentration in breast muscle from line S compared with line D (Figure 3A). Meanwhile, 77 metabolites had a higher concentration, and 38 had a lower concentration in thigh muscle from line S compared with line D (Figure 3B).

### 2.5. Pathway Enrichment Analysis

**Figure 4:**
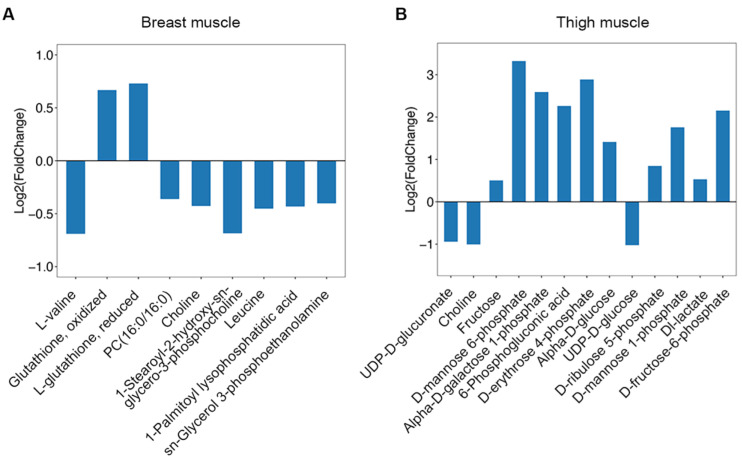
The change of metabolites enriched in pathways from different muscles between line S and line D chickens. (**A**) The concentration changes of metabolites involved in enriched pathways in breast muscle. (**B**) The concentration changes of metabolites involved in enriched pathways in thigh muscle. Log2(Fold Change) > 0 indicates an increased concentration in line S, and log2(Fold Change) < 0 indicates the decreased concentration in line S.

The tested compounds were input into MetaboAnalyst 4.0, and latent pathways were enriched based on the KEGG database. In breast muscle, the concentrations of valine and leucine decreased, and the levels of sn-glycerol 3-phosphoethanolamine, choline, PC(16:0/16:0), 1-palmitoyl lysophosphatidic acid, 1-stearoyl-2-hydroxy-sn-glycero-3-phosphocholine declined; while the levels of L-glutathione, reduced and glutathione, oxidized increased (Figure 4A). Therefore, valine, leucine, and isoleucine biosynthesis, glycerophospholipid metabolism, and glutathione metabolism were significantly changed (*p* < 0.05) (Table 1). In thigh muscle, the increased levels of alpha-D-glucose, alpha-D-galactose 1-phosphate, fructose, D-mannose 6-phosphate, D-mannose 1-phosphate and D-fructose-6-phosphate indicated that amino sugar and nucleotide sugar metabolism changed. The concentrations of 6-phosphogluconic acid, D-ribulose 5-phosphate, D-erythrose 4-phosphate were found to have increased, and the levels of UDP-D-glucose, UDP-D-glucuronate and choline decreased (Figure 4B). These results suggest that ascorbate and aldarate metabolism, the pentose phosphate pathway, pentose and glucuronate interconversions, fructose and mannose metabolism, and glycerophospholipid metabolism were transformed in thigh muscle from line S (Table 1).

## 3. Discussion

Slow-growth chicken breeds generally display lower body weight than fast-growth populations during the same growth period. However, indigenous chickens are favored by consumers, particularly in China and France, where they represent 41.67% of the market share in China and 24% in France [26]. Unlike several commercial chicken breeds that have almost attained their maximum growth capability, native populations still retain massive developmental potential. The Guangxi Partridge chicken is an indigenous species that possesses several excellent traits, which has been differentiated into two lines by selective breeding for four generations. Our previous study found that the body weight of line S was 200 g heavier than unselected chickens on average at age of 90 days, and no other health problems were exhibited [24]. Therefore, this population was a good model for exploring the impact of selective breeding on meat quality.

Color (i.e., L*, a*, and b*), pH, shear force, and drip loss of skeletal muscles are important indices for consumers, which were quantified in breast and thigh muscles between different line chickens in the present study. Here, L* and b* increased and a* decreased in breast and thigh muscles of line S chickens, although only L* and b* showed statistically significant differences (*p* < 0.05). pH was in the normal range. pH_15_ and pH_u_ decreased in breast muscle of line S chickens (*p* > 0.05), while pH_15_ and pH_u_ in thigh muscle was significantly reduced (*p* < 0.05). Weng et al. found similar results in that L* improved significantly and a* was reduced in thigh muscle of fast-growth chickens, and breast and thigh muscles from fast-growth birds always showed a low pH [27].

Shear force represents the tenderness of muscle, which declined in breast and thigh muscles of line S birds, although it was significantly different in breast muscle. Several studies found that slow-growth chickens displayed considerable improvement in shear force in breast and thigh muscles [28,29]. Although others reported that breast muscle in slow-growth chickens was more tender than broilers; the different results in these studies may be attributed to strains, rearing conditions, or other factors [30,31].

Drip loss is an indispensable edible attribution of meat, and drip loss of breast and thigh muscles all increased in line S chickens in our study. Chartrin et al. found that overfed ducks exhibited high cooking loss and more flavor, which may result from the increased level of fat in muscle, and others revealed that the level of drip loss in fast-growth chickens was elevated [32,33]. In contrast with younger ducks, pH, tenderness, and juice loss decreased at 15 w of age, which should be related to change in myofiber size [34]. Reduced pH, high L*, and drip loss are generally related to increased oxidative stress [35,36]. We collected the RNA-seq data that was performed with the same skeletal muscles in this study [24] and found that the expression of several Heat Shock Protein (HSP) genes was significantly upregulated in breast and thigh muscles in line S chickens (Figure S2), and upregulation of HSP genes could be associated with oxidative stress [37,38]. We speculated that intensive selective breeding contributed to generation of oxidative stress and further changed the quality of breast and thigh muscles, which was reflected by alteration of color, pH, shear force, and drip loss.

To clarify the effect of artificial selection on skeletal muscles, the LC-MS/MS method was adopted to detect differentiated metabolites in selected chickens. Here, 151 and 115 differential metabolites were identified in the breast and thigh muscles from two groups, respectively. First, the concentration of Dl-lactate accumulated in thigh muscle from line S (Figure 4B and Table S2), which revealed the confusion over the significant decline in pH_15_. The valine, leucine, and isoleucine biosynthesis pathway was changed in breast muscle by KEGG analysis, which performs an essential function in breast muscle development under artificial selection. Valine, leucine, and isoleucine are branched-chain amino acids (BCAAs), and leucine is not only a key precursor substance for the synthesis of muscle protein, it also regulates intracellular signal pathways of protein formation. It is now known that a low concentration of BCAAs is required for optimal development during chicken growth [39]. Tomonaga et al. compared different developmental periods of chickens and found that the levels of BCAAs in muscle were reduced along with muscle development [40], and the concentrations of L-valine and leucine were declined in line S (Figure 4A) in this study, which indicated that muscle growth might consume more BCAAs.

In addition, glycerophospholipid metabolism was the second significantly changed pathway identified in this study, and it has been studied widely in muscle development for components of cellular membranes. To acquire knowledge of muscle wasting, Senoo et al. adopted a metabolomic method to explore the glycerophospholipid profile of skeletal muscle in a muscular dystrophy model using a denervation murine, and they found an apparent alteration of glycerophospholipids [41]. Jiang et al. reported that whole-body vibration, which is a special exercise for elderly people, had an impact on glycerophospholipid metabolism to reduce lipid deposition in aging muscles [42].

The concentrations of sn-glycerol 3-phosphoethanolamine, choline, PC(16:0/16:0), 1-palmitoyl lysophosphatidic acid, and 1-stearoyl-2-hydroxy-sn-glycero-3-phosphocholine were lower in breast muscle in line S (Figure 4A and Table S1), and they were involved in the biosynthesis of phosphatidylethanolamine (PE) and phosphatidylcholine (PC). Glutathione metabolism changed considerably in the present study that both of the concentrations of glutathione, oxidized and L-glutathione, reduced, were elevated in line S (Figure 4A and Table S1). Glutathione is an antioxidant composed of cysteine, glutamic acid, and glycine, and this pathway suggested that oxidative stress was initiated in line S chickens was due to selection. Usually, it is thought that chickens with slow growth show better flavor, meat quality, and anti-oxidation ability [43,44,45]. Turinsky et al. found that dissimilar concentrations of several free amino acids were identified in different types of myofibers; furthermore, increased concentrations of leucine and glutamic acid and a reduced level of glutamine and glycine were observed in denervation atrophied muscles [46]. Some studies proved that the concentration of glutathione declined in atrophic muscles due to reactive oxygen species, although Tachibana et al. claimed that glutathione was highly detected in the denervation-induced muscle atrophy model; however, they speculated that the result was attributed to the brief duration of denervation [47,48,49]. Zhang et al. found that glycerophospholipid metabolism and glutathione metabolism were significantly changed in breast muscle between slow-growth and fast-growth chickens, which were considered important functions in meat flavor [50]. From all of the above, we fully hypothesize that the improved body weight of line S chickens depended on alteration of valine, leucine, and isoleucine biosynthesis and glycerophospholipid metabolism, but the accompanying oxidative injury acquired glutathione metabolism to release.

Compared with the three pathways identified in breast muscle, we screened six pathways in thigh muscle. Amino sugar and nucleotide sugar metabolism was the most significant identified pathway (*p* = 9.33 × 10^−9^), which was exhibited by the increased levels of alpha-D-glucose, alpha-D-galactose 1-phosphate, fructose, D-mannose 6-phosphate, D-mannose 1-phosphate, and D-fructose-6-phosphate in line S chickens (Figure 4B and Table S2). Huo et al. compared fast-growth and slow-growth chicken breeds, and they found high concentrations of glycogen, glucose, glucose-6-phosphate, lactic acid, and glycolytic potential in fast-growth fowls [15]. Dark meat is a serious problem in the beef industry, which was determined by reduced glycolytic potential and a high level of several mitochondrial enzymes [51]. Cônsolo et al. used metabolomics to screen different substances between normal and dark meat, and they found that amino sugar and nucleotide sugar metabolism changed dramatically; this suggested a strong correlation between glycolysis and amino sugar and nucleotide sugar metabolism [52].

Combined with the elevated concentration of Dl-lactate, we concluded that glycolysis developed in thigh muscle. Bawa et al. proved that reduced glycolytic ability decreased the size of brain and muscle tissues in Drosophila, and others have claimed that dysfunction of glycolytic metabolism resulted in a decline in the cardiomyocyte cell cycle, cell number, and heart size [53,54]. Certainly, the pentose phosphate pathway, a branch of glycolysis, could catalyze glucose-6-phosphate to D-fructose-6-phosphate, which was identically changed. The pentose phosphate pathway diverts into two directions: the oxidative branch and the nonoxidative branch, both of which produce NAPDH, which is a pivotal antioxidant that is involved in the biogenesis of several lipids [55].

We found that the levels of 6-phosphogluconic acid, D-ribulose 5-phosphate, and D-erythrose 4-phosphate increased, although the concentration of D-xylulose 5-phosphate, which was a product of D-ribulose 5-phosphate, was statistically unchanged in thigh muscle from line S chickens (Figure 4B and Table S2). 6-phosphogluconic acid, D-ribulose 5-phosphate, and D-xylulose 5-phosphate were attached to the oxidative branch, and D-erythrose 4-phosphate and D-fructose 6-phosphate were end-products of the nonoxidative channel. This suggested that the two branches both performed functions in the thigh muscle, and the nonoxidative pattern exerted more effects on thigh muscle than the oxidative pattern. However, Wagner et al. explored changes in the pentose phosphate pathway in the remodeling of skeletal muscle by detecting the level of related enzymes. They determined that the expression of oxidative enzymes increased more than nonoxidative enzymes, which suggested that the oxidative branch of the pentose phosphate pathway functioned in myogenesis [56]. The dissimilarity with the present study may be because the remodeling of skeletal muscle depended on a toxic drug, which intensified the requirement for NAPDH; this resulted in switching to the oxidative pattern in the pentose phosphate pathway.

Ascorbate and aldarate metabolism was the second identified pathway in thigh muscle from line S compared with line D, which was represented by decreased concentrations of UDP-D-glucose and UDP-D-glucuronate and a high concentration of L-ascorbate (Figure 4B and Table S2). L-ascorbate is a strong antioxidant that is involved in the synthesis of many important compounds. Ascorbate and aldarate metabolism was upregulated in different types of oxidative injuries, and this disorder could be corrected using the appropriate drugs [57,58]. Nasr et al. compared the oxidative status of different chicken breeds, suggested that the serum from individuals with high body weight showed a significantly increased level of malondialdehyde (MDA) [35]; others claimed that the concentration of glutathione peroxidase (GPX) in breast muscle of fast-growth birds was considerably elevated [45]. These studies indicated that a rapid growth rate contributed to high oxidative stress, which explained the change in ascorbate and aldarate metabolism. Additionally, ascorbic acid 2-phosphate, which is a derivative of L-ascorbate, was found to facilitate myoblast differentiation, which showed the potential for L-ascorbate to promote myogenesis [59].

Conspicuously, UDP-D-glucose and UDP-D-glucuronate combined with a reduced concentration of D-ribulose 5-phosphate was integrated into the pentose and glucuronate interconversions pathway, which was also altered significantly in the present study, and this pathway functioned in the detoxification and excretion of metabolites [60,61]. Wang et al. screened differentiated metabolites to explore the effect of the density of chickens during rearing on chicken performance, and they found that the pentose and glucuronate interconversions pathway and the pentose phosphate pathway had the most significant influence in high density groups of birds [62]. Various studies suggested that the two pathways were roughly regarded to the kinds of aberrant physiological conditions exhibited [61,63,64]. Therefore, these pathways may have combined to reduce oxidative stress that resulted from selective breeding for greater meat yield.

In addition, fructose and mannose metabolism was profoundly changed in thigh muscle, which suggested that it played an indispensable role in skeletal muscle under selection. To find a therapeutic remedy for atrophy of denervated muscles, Zhou et al. found that traditional Chinese medicine restored puniness of skeletal muscle, and fructose and mannose metabolism, galactose metabolism, and glycerolipid metabolism were upregulated in recovered tissues. This indicated that activation of these pathways reduced inflammation from denervation and improved the function of skeletal muscle [65]. Salau et al. explored oxidative injury in the pancreas and found that ascorbate and aldarate metabolism, pentose and glucuronate interconversions, fructose and mannose metabolism, and amino sugar and nucleotide sugar metabolism were influenced, which indicated that these pathways functioned in unison in oxidative stress [58]. A significant change in the glycerophospholipid metabolism pathway was also identified in thigh muscle, which was screened in breast muscle, which may perform similar functions in thigh muscle. Michel et al. reported that food supplemented with choline improved the birth weight of cattle by lowering DNA methylation [66]. The concentration of choline decreased significantly in both breast and thigh muscles in line S chickens (Figure 4), which suggested that selection accelerated the consumption of choline and promoted muscle development. Therefore, nutritionists could design more effective chicken feeds to improve growth rate by altering glycerophospholipid metabolism.

**Figure 5:**
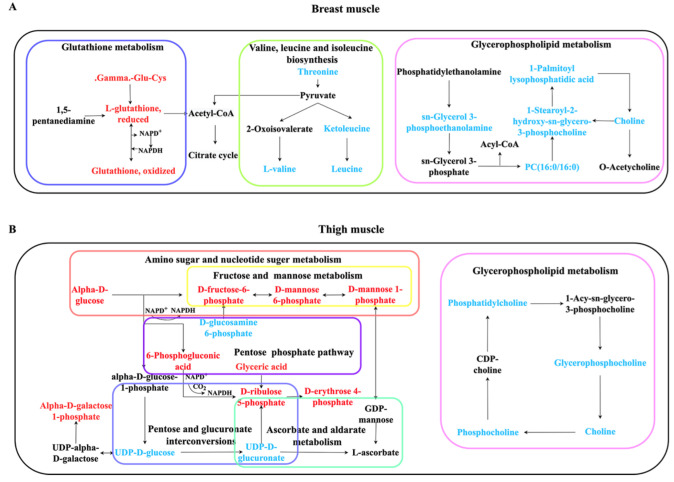
Schematic sketch of enriched KEGG pathways in breast muscle (**A**) and thigh muscle (**B**). Red indicates that the concentration of metabolites is increased, and blue indicates a decrease. Black metabolites are related metabolites or pathways; however, they are not statistically significant in the present study. Boxes with different color indicate divergent pathways.

To better understand the mechanism of selective breeding on meat quality, we drew a schematic diagram using enriched pathways from breast and thigh muscles. These pathways interacted to shape complicated networks (Figure 5).

## 4. Experimental Design

### 4.1. Animals

Two lines of Guangxi Partridge chicken were raised at the Guangxi Fufeng Agricultural and Animal Husbandry Group Co., Ltd. (Nanning, China) for 90 days. Chickens were fed on a basic diet with drinking water. The same diet was supported for all chickens throughout the trial, employing a starter diet (21% protein) for chickens 0 to 3 weeks of age, a grower diet (19% protein) for birds 4 to 6 weeks of age, and a finisher diet (17% protein) for birds 7 to 13 weeks of age. For line S selection, the top 3% of roosters and top 20% of hens were selected for body weight for breeding. Line D was selected for higher egg production, while its body weight was maintained as the initial population. After four generations, 10 individuals were randomly chosen from each line and were harvested to detach breast and thigh muscles at the age of 90 days. The body weights of 10 line S cockerels were about 1.724 ± 0.128 kg, and the line D was about 1.509 ± 0.084 kg for 90 days [24]. Then, eight individuals were selected to perform further metabolome research. After breast and thigh muscles were isolated from the chicken, a piece of tissue was scissored, plugged into 2 mL RNase-free tubes and immediately placed into liquid nitrogen. Then, the rest of the muscle tissue was used to measure meat quality. All procedures were approved by the Nanjing Agricultural University Animal Ethical and Welfare Committee (SYXK2011-0036).

### 4.2. Assay of Meat Quality

Meat color was detected after slaughter, which was evaluated by a colorimeter (Minolta CR-10, Konica Minolta, Tokyo, Japan) with the CIELAB system (L*: Lightness; a*: Redness; b*: Yellowness). The probe of the pH meter (F2 pH meter, Mettler Toledo, America) was inserted into the muscle to a depth of 1 cm to acquire a pH value at 15 min (pH_15_) and 24 h (pH_u_) after slaughter. L*, a*, b*, and pH were measured in triplicate at three points for each tissue, and the average value was recorded. Muscle was cut into 1 cm^3^ samples, weighed, and suspended in a sealed box at 4 °C in a refrigerator. After 24 h, we reweighed these tissues, and the difference in weights for each tissue was considered as drip loss.

### 4.3. Sample Preparation for Metabolomics

Skeletal muscle tissues were taken from a −80 °C refrigerator and were dissected in dry ice (~80 mg) into an Eppendorf tube (2 mL). The tissues were homogenized with 200 μL water and five ceramic beads with FastPrep-24 5G homogenizer. The time of homogenization was 60 s, the speed was 6.0 M/S, and it was repeated 3 times. Methanol/acetonitrile (1:1, *v*/*v*) was added to the mixed solution for further extraction of metabolites. The blend was centrifuged for 15 min at 14,000× *g* and 4 °C. The supernatant was dried in a vacuum centrifuge. Samples were redissolved in 100 μL acetonitrile/water (1:1, *v*/*v*) for the LC-MS/MS analysis.

### 4.4. Measurement of Metabolites

Detection of metabolites was determined by an UHPLC (1290 Infinity LC, Agilent Technologies) coupled to a quadrupole time-of-flight (AB Sciex TripleTOF 6600) from Shanghai Applied Protein Technology Co., Ltd. (Shanghai, China). Samples were moved into ACQUITY UPLC BEH 1.7 μm column (Waters, ACQUITY UPLC BEH Amide) to perform the HILIC separation. An ESI source was used in the positive and negative ionization mode. In both ESI modes, the mobile phase contained 25 mM ammonium acetate and 25 mM ammonium hydroxide in water (A) or acetonitrile (B). The gradient was 85% B for 1 min and was linearly reduced to 65% for 11 min, and then was reduced to 40% for 0.1 min and kept for 4 min, and then increased to 85% for 0.1 min, with re-equilibration for 5 min. For RPLC separation, an ACQUITY UPLC HSS T3 1.8 μm column was used. Water with 0.1% formic acid (A) and acetonitrile with 0.1% formic acid (B) were maintained in mobile phase in positive mode, and 0.5 mM ammonium fluoride was added to both water (A) or acetonitrile (B) in the mobile phase in negative mode. The setup was as follows: 1% B for 1.5 min, elevated to 99% for 11.5 min, remaining for 3.5 min, decreased to 1% for 0.1 min, and 3.4 min for re-equilibration. The flow rate was 0.3 mL/min, and the column was maintained at 25 °C. Then, a 2 μL aliquot of each sample was injected.

The ESI conditions were set as follows: Ion Source Gas1 at 60 psi; Ion Source Gas2 at 60 psi; Curtain Gas at 30 psi; Source temperature, 600 °C; IonSpray Voltage Floating, ±5500 V. in MS period; the machine was set to gain over the m/z range of 60–1000 Da; and the accumulation time for TOF MS scan was set at 0.2 s/spectra. In MS/MS time, the instrument was set to gain over the m/z range of 25–1000 Da, and the accumulation time for the TOF MS scan was set at 0.05 s/spectra. The parameters for the ion scan were set as follows: collision energy at 35 V with ±15 eV, declustering potential of 60 V (+) and 60 V (−), excluding isotopes within 4 Da, and candidate ions to monitor per cycle: 10.

### 4.5. Analysis of Metabolomic Data

The identification, filtering, and alignment of peaks were performed to acquire mass-to-charge ratio, retention time, and peak area based on positive and negative ion models. The raw data were transformed into mzXML format by ProteoWizard and the XCMS project was employed for peak alignment, retention time correction as well as peak area extraction. The condition of XCMS was set as follows: centwave settings for feature detection (Δm/z = 25 ppm, peakwidth = c (10, 60)); obi-warp settings for retention time correction (profStep = 1); and conditions including minfrac = 0.5, bw = 5 and mzwid = 0.025 for chromatogram alignment. Peaks of metabolites were matched with the mzCloud database (https://www.mzcloud.org/, accessed on 6 September 2021); Thermo mzVault (version 2.3) was used to create a mass spectral library and Tracefinder (verison 4.1) was adopted for quantification; Glycopep Masslist software was employed for glycopeptide analysis with a default glycan library (including 340 glycans).

The log transformation (base 10) was applied to normalize the data. Data scaling was performed using mean-centered and was divided by the standard deviation of each variable. Principal component analysis (PCA) and orthogonal projection to latent structures-discriminant analysis (OPLS-DA) were used to reveal differences in metabolites between the two chicken lines. Potential metabolites were screened based on the variable importance in the projection (VIP) value gained from OPLS-DA and the *p* value from Student’s t test. Metabolites with a VIP value greater than 1 and a *p* value smaller than 0.05 were considered statistically significant. These tests were performed using R software and MetaboAnalyst (V5.0) (https://www.metaboanalyst.ca/ accessed on 12 November 2021).

### 4.6. RNA Extraction and Sequencing

Three individuals were chosen from each eight samples for each line for RNA sequencing [24]. RNA was extracted from Breast and thigh muscles by Trizol reagent (Invitrogen, Carlsbad, CA, USA). The integrity and quality of RNA were measured, enriched with Oligo beads, reversed into cDNA, and sequenced using Illumina Novaseq 6000 by Gene Denovo Biotechnology Co. (Guangzhou, China).

### 4.7. Statistical Analysis

The results were analyzed using Prism software (v6.0). Data are shown as the mean ± standard deviation (SD). If the *p* value is less than or equal to 0.05, the results are considered statistically significant.

## 5. Conclusions

We determined that selective breeding improved the performance of Guangxi Partridge chickens and that this breeding induced oxidative stress and changed the quality of breast and thigh muscles, which included a change in meat color, pH, shear force, and drip loss. LC-MS/MS-based metabolomics showed that valine, leucine, and isoleucine biosynthesis, glycerophospholipid metabolism, and glutathione metabolism changed significantly in breast muscle, while amino sugar and nucleotide sugar metabolism, ascorbate and aldarate metabolism, the pentose phosphate pathway, pentose and glucuronate interconversions, fructose and mannose metabolism, and glycerophospholipid metabolism changed in thigh muscle. Glycerophospholipid metabolism functioned synchronously in breast and thigh muscles, which suggested a conserved role in the development of skeletal muscle. Although different therapies were needed to deal with oxidative stress from the selection in different muscle tissues, glutathione metabolism performed an anti-oxidative effect in breast muscle, and several pathways responded to oxidative injury in thigh muscle.

## Figures and Tables

**Table 1:** KEGG-enriched analysis of differentiated metabolites from breast and thigh muscles of line S and line D chickens.

Tissue	Pathway ID ^1^	Pathway Name ^2^	N ^3^	*p* Value
Breast muscle	ko00290	Valine, leucine and isoleucine biosynthesis	3	0.00132
	ko00564	Glycerophospholipid metabolism	5	0.00339
	ko00480	Glutathione metabolism	4	0.00894
Thigh muscle	ko00520	Amino sugar and nucleotide sugar metabolism	11	9.33 × 10^−9^
	ko00053	Ascorbate and aldarate metabolism	3	0.00371
	ko00030	Pentose phosphate pathway	4	0.00542
	ko00040	Pentose and glucuronate interconversions	3	0.02082
	ko00051	Fructose and mannose metabolism	3	0.02082
	ko00564	Glycerophospholipid metabolism	4	0.02806

^1^ Pathway ID, ID of enriched KEGG pathway; ^2^ Pathway name, name of KEGG pathway; ^3^ N, number of KEGG pathways containing differentiated metabolites.

## Data Availability

Not applicable.

## Supplementary Materials

The following supporting information can be downloaded at: https://www.mdpi.com/article/10.3390/metabo12050367/s1, Figure S1: The heatmap of correlations between all samples. Figure S2: The relative expression of several genes from RNA-seq. Table S1: Differentiated metabolites in breast muscle between line S and line D. Table S2: Differentiated metabolites in thigh muscle between line S and line D.
