# Supplementary material for: Untargeted Metabolomics Reveals the Effect of Selective Breeding on the Quality of Chicken Meat
Source: Metabolites. 2022 Apr 19;12(5):367. doi: 10.3390/metabo12050367 (PMC9144515; doi:10.3390/metabo12050367)
Supplement: Supplementary file 1 [file metabolites-12-00367-s001.zip › Table S1. Differentiated metabolites in breast muscle between line S and line D.pdf]

**Table S1**. Differentiated metabolites in breast muscle between line S and line D.

| Metabolite name | log2(FC) | p-value | VIP-value |
|---|---|---|---|
| Sm d34:1 | -0.8008 | 0.0001 | 2.6478 |
| 1-Myristoyl-2-stearoyl-sn-glycero-3-phosphocholine | -0.5042 | 0.0001 | 2.6263 |
| Ethyl vanillate | -0.6497 | 0.0002 | 2.6122 |
| Pc(16:1e/17-hdohe) | -0.3903 | 0.0002 | 2.5784 |
| Nocardamine | -0.9716 | 0.0003 | 2.5163 |
| Guanosine diphosphate mannose | 0.7519 | 0.0003 | 2.4935 |
| 3,8''-Biapigenin | 1.2187 | 0.0007 | 2.4345 |
| 1,2-Diamino-2-methylpropane | -0.7038 | 0.0008 | 2.3853 |
| Juglone | 0.5019 | 0.0010 | 2.3645 |
| Quinolin-2-ol | 0.8474 | 0.0015 | 2.3532 |
| Isobutyryl-l-carnitine | 1.0875 | 0.0017 | 2.3696 |
| Gossypol | 0.4249 | 0.0019 | 2.3150 |
| (2-Aminoethoxy)[2-[docosa-4.7.10.13.16.19-hexaenoyloxy]-3-[octadeca-1.9-dien-1-yloxy]propoxy]phosphinic acid | -0.7639 | 0.0020 | 2.2898 |
| Phenol, 5-(1,1-dimethylheptyl)-2-[(1r,2r,5r)-5-hydroxy-2-(3-hydroxypropyl)cyclohexyl]- | -0.5429 | 0.0021 | 2.2493 |
| L-valine | -0.6902 | 0.0022 | 2.2538 |
| Glutathione, oxidized | 0.6675 | 0.0025 | 2.2272 |
| 2'-Deoxycytidine 5'-diphosphate | 1.0892 | 0.0025 | 2.2435 |
| L-lysine | -0.9819 | 0.0026 | 2.2481 |
| Flavin adenine dinucleotide (FAD) | 0.9953 | 0.0028 | 2.2104 |
| N-(2-furoyl)glycine | 0.2506 | 0.0029 | 2.2379 |
| Gly-His | -0.8674 | 0.0030 | 2.2324 |
| Sm d34:2 | -0.4962 | 0.0032 | 2.2619 |
| Guanidinopropionic acid | 0.4303 | 0.0034 | 2.2252 |
| 2'-O-methylinosine | 0.5372 | 0.0034 | 2.2013 |
| L-glutathione, reduced | 0.7294 | 0.0036 | 2.1774 |
| Clomazon | 0.5623 | 0.0038 | 2.1975 |
| Harpagoside | 0.6184 | 0.0040 | 2.2020 |
| 1-Palmitoyl-2-oleoyl-phosphatidylglycerol | -0.4728 | 0.0044 | 2.1760 |
| PC(16:0/16:0) | -0.3610 | 0.0045 | 2.1755 |
| 2-Phenylpiperidine-2-acetamide | 0.4276 | 0.0048 | 2.1527 |
| Muramic acid | 0.2827 | 0.0048 | 2.1291 |
| Pro-his | -0.7239 | 0.0051 | 2.1316 |
| Isoproterenol | -0.2577 | 0.0051 | 2.1447 |
| Taurochenodeoxycholate | -3.3965 | 0.0054 | 2.1350 |
| Tauroursodeoxycholic acid | -2.9129 | 0.0062 | 2.1054 |
| His-Gly | -0.6895 | 0.0064 | 2.1061 |
| Poncirin | 0.4976 | 0.0065 | 2.0932 |

| | | | |
|---|---|---|---|
| Taurodeoxycholic acid | -2.6473 | 0.0067 | 2.0846 |
| Sphingosine | -0.8549 | 0.0069 | 2.0749 |
| 1-Stearoyl-2-linoleoyl-sn-glycero-3-phospho-l-serine | 0.4754 | 0.0070 | 2.0600 |
| 3,5-Di-tert-butyl-4-hydroxybenzoic acid | -0.2301 | 0.0071 | 2.0795 |
| 4-Hydroxyisoleucine | 0.8614 | 0.0071 | 2.1045 |
| Ala-Glu-Arg | -0.4785 | 0.0074 | 2.0734 |
| 10,12-Tricosadiynoic acid | -2.0325 | 0.0088 | 2.0356 |
| 4-Piperidinecarboxamide | -0.7280 | 0.0097 | 2.0564 |
| Isovaleryl-l-carnitine | 1.9586 | 0.0107 | 2.0211 |
| Ketoleucine | -0.9372 | 0.0108 | 1.9824 |
| Aspartic acid | -0.3802 | 0.0108 | 2.0197 |
| Choline | -0.4266 | 0.0110 | 1.9540 |
| Licoricesaponin h2 | 1.2327 | 0.0113 | 2.0065 |
| Cer[ns] d34:1 | -0.6995 | 0.0115 | 2.0022 |
| (2-Aminoethoxy)[2-[docosa-4.7.10.13.16.19-hexaenoyloxy]-3-[hexadec-1-en-1-yloxy]propoxy]phosphinic acid | -0.4820 | 0.0116 | 1.9932 |
| L-citrulline | -0.3882 | 0.0117 | 2.0263 |
| 2-Naphthalenesulfonic acid | 0.9751 | 0.0125 | 1.9457 |
| 1h-Imidazo[4,5-c]pyridine-6-carboxylic acid, 1-[[4-(dimethylamino)-3-methylphenyl]methyl]-5-(2,2-diphenylacetyl)-4,5,6,7-tetrahydro-, (6s)- | -0.7414 | 0.0126 | 1.9292 |
| Propanoic acid, 2-[[4-[2-[[(cyclohexylamino)carbonyl](4-cyclohexylbutyl)amino]ethyl]phenyl]thio]-2-methyl- | -1.6134 | 0.0127 | 1.9679 |
| Trans-4-(aminomethyl)cyclohexanecarboxylic acid | 0.3734 | 0.0129 | 1.9178 |
| Zanamivir | -1.8380 | 0.0133 | 1.9470 |
| Benzeneheptanol, 3-hydroxy-4-[(1r,3s)-3-hydroxycyclohexyl]-.eta.,.eta.-dimethyl-, rel- | 2.7693 | 0.0135 | 1.8969 |
| 5-Methylcytidine | 0.3666 | 0.0137 | 1.9711 |
| 2-Arachidonoyl-1-palmitoyl-sn-glycero-3-phosphoethanolamine | -0.3308 | 0.0142 | 1.9642 |
| Anthranilic acid | -0.7109 | 0.0143 | 1.9363 |
| .Gamma.-Glu-Cys | 0.7414 | 0.0144 | 1.9303 |
| Indolelactic acid | -0.4015 | 0.0145 | 1.9131 |
| D-erythro-imidazolylglycerol phosphate | -0.5768 | 0.0146 | 1.9486 |
| Cholesteryl sulfate | -0.6023 | 0.0162 | 1.9348 |
| Ne-acetyllysine | -0.5992 | 0.0163 | 1.8737 |
| Ser-Tyr | 0.5433 | 0.0166 | 1.8760 |
| Trigonelline | 0.7858 | 0.0167 | 1.9298 |
| Glutamine | -0.3364 | 0.0171 | 1.8715 |
| Pc(18:0e/20-hdohe) | -0.5108 | 0.0175 | 1.9010 |
| N-phenylacetyl-l-prolylglycine ethyl ester | -0.7339 | 0.0183 | 1.8632 |
| N-(4-piperidinyl) acetamide | -0.7106 | 0.0186 | 1.8764 |
| Swietenocoumarin b | -0.6399 | 0.0188 | 1.9058 |

| | | | |
|---|---|---|---|
| Fusidic acid | -1.5097 | 0.0200 | 1.8658 |
| Ser-Gly | -0.9106 | 0.0202 | 1.8691 |
| Asiatic acid | -1.5782 | 0.0203 | 1.8615 |
| Adenosine 5'-monophosphate | -0.3994 | 0.0205 | 1.8766 |
| 2'-Hydroxy-3,4,6'-trimethoxychalcone | -0.2218 | 0.0213 | 1.8875 |
| N-acetyl-s-geranylgeranyl-l-cysteine | -0.7825 | 0.0224 | 1.7827 |
| 2-Acetyl-6-[(3-butanoyl-2,4,6-trihydroxy-5-methylphenyl)methyl]-3,5-dihydroxy-4,4-dimethylcyclohexa-2,5-dien-1-one | 0.4938 | 0.0226 | 1.7875 |
| D-myo-inositol-1,4,5-triphosphate | 0.4281 | 0.0228 | 1.7933 |
| 1h-Indazole-3-carboxamide, n-[(1s)-1-(aminocarbonyl)-2-methylpropyl]-1-(cyclohexylmethyl)- | -0.5247 | 0.0235 | 1.7807 |
| S-adenosyl-l-methionine | -0.8833 | 0.0239 | 1.7932 |
| 4-Hydroxy-l-phenylglycine | -1.0994 | 0.0240 | 1.8309 |
| 1-Naphthol .beta.-d-glucuronide | 0.4101 | 0.0244 | 1.7555 |
| N.omega.-propyl-l-arginine | -0.5939 | 0.0245 | 1.8179 |
| Zoledronic acid | -0.4086 | 0.0248 | 1.8256 |
| Isoginkgetin | 0.8549 | 0.0248 | 1.7572 |
| N6,N6,N6-Trimethyl-L-lysine | -0.5824 | 0.0249 | 1.7883 |
| Crocin | -0.6158 | 0.0252 | 1.8374 |
| 2s-Amino-4-phosphonobutyric acid | -0.6223 | 0.0253 | 1.8072 |
| 1-Stearoyl-2-hydroxy-sn-glycero-3-phosphocholine | -0.6847 | 0.0254 | 1.7550 |
| 1-(1z-Hexadecenyl)-sn-glycero-3-phosphocholine | -0.7752 | 0.0267 | 1.7519 |
| Gly-Gly | -0.5953 | 0.0268 | 1.7983 |
| Theophylline | 0.2439 | 0.0286 | 1.7249 |
| Pi 36:4 | -0.4135 | 0.0287 | 1.7830 |
| 3'-O-methylguanosine | 0.2749 | 0.0287 | 1.7650 |
| 1,5-Pentanediamine | -0.4758 | 0.0289 | 1.7097 |
| Octyl-3,5-di-tert-butyl-4-hydroxyhydrocinnamate | -1.2912 | 0.0296 | 1.6973 |
| 2'-O-methylcytidine | 0.2126 | 0.0305 | 1.7511 |
| Leucine | -0.4522 | 0.0308 | 1.6960 |
| 1-Stearoyl-rac-glycerol | -0.6734 | 0.0310 | 1.7119 |
| Eurycomalactone | -1.6805 | 0.0311 | 1.7378 |
| Dihydrothymine | -0.3636 | 0.0312 | 1.7306 |
| 1-Myristoyl-2-hydroxy-sn-glycero-3-phosphoethanolamine | -0.6447 | 0.0314 | 1.7021 |
| Alpha-tocopherol (Vitamin E) | -0.7350 | 0.0315 | 1.7255 |
| Desisopropyldisopyramide | 0.5117 | 0.0316 | 1.7239 |
| 3'-O-methylcytidine | 0.5583 | 0.0318 | 1.7187 |
| 1,2-Dithiolane-3-pentanamide, n,n'-1,3-propanediylbis- | -0.4631 | 0.0319 | 1.7922 |
| 3,4-Dichlorophenol | -0.4548 | 0.0322 | 1.7513 |
| Guanosine 5'-diphospho-.alpha.-d-mannose | 0.4677 | 0.0327 | 1.6795 |
| N-formyl-l-methionine | -0.5811 | 0.0332 | 1.7094 |
| Ethylenediaminetetraacetic acid | 0.8888 | 0.0347 | 1.6955 |

| | | | |
|---|---|---|---|
| 4,2'-Dihydroxy-3,4',6'-trimethoxychalcone | 0.4283 | 0.0350 | 1.6763 |
| Arg-Cys | 0.3888 | 0.0355 | 1.6981 |
| 7-Oxocholesterol | 1.4103 | 0.0356 | 1.7234 |
| 1-Palmitoyl lysophosphatidic acid | -0.4317 | 0.0357 | 1.6756 |
| Adp-ribose | 0.4169 | 0.0358 | 1.7170 |
| Gly-Asn | -0.5192 | 0.0362 | 1.7061 |
| Octadecanoic acid | -0.2876 | 0.0363 | 1.7235 |
| N-acetylserine | -0.4231 | 0.0364 | 1.6596 |
| 3- Quinolinecarboxylic acid, 1,4,5,6,7,8-hexahydro-4-(3-hydroxyphenyl)-7-(2- methoxyphenyl)-2-methyl-5-oxo-, 2-methoxyethyl ester | -0.5860 | 0.0368 | 1.7184 |
| Pentaerythritol tetrakis(3,5-di-tert-butyl-4-hydroxyhydrocinnamate) | -0.4416 | 0.0375 | 1.7246 |
| Hypoxanthine | -0.4810 | 0.0382 | 1.7033 |
| Pro-Gly-Lys | -0.2598 | 0.0383 | 1.6833 |
| L-glutamine | -0.3861 | 0.0389 | 1.6984 |
| Threonine | -0.5833 | 0.0402 | 1.6441 |
| N-palmitoyl-d-sphingosine | -0.5682 | 0.0407 | 1.6982 |
| Dibucaine | 0.6345 | 0.0408 | 1.6396 |
| Salmeterol | -0.6220 | 0.0412 | 1.6426 |
| Acetylcarnitine | 0.2791 | 0.0413 | 1.6646 |
| Pc(16:0e/10-hdohe) | -0.3876 | 0.0415 | 1.6756 |
| Pc(18:1e/14,15-eet) | -0.4852 | 0.0417 | 1.6737 |
| Flavone base + 3o, c-pen, c-pen | 0.7299 | 0.0418 | 1.6172 |
| Goitrin | -0.9789 | 0.0422 | 1.6840 |
| Condelphine | -0.5175 | 0.0423 | 1.6300 |
| Lisinopril (8r,s)-diketopiperazine | -0.1426 | 0.0425 | 1.6850 |
| 2,4-Dichlorobenzoic acid | 0.4664 | 0.0426 | 1.6435 |
| 1-(1z-Octadecenyl)-sn-glycero-3-phosphocholine | -0.7650 | 0.0431 | 1.5915 |
| Hyodeoxycholic acid | -0.7740 | 0.0431 | 1.6749 |
| L-tryptophanamide | 1.3548 | 0.0450 | 1.6475 |
| N-alpha-acetyl-l-lysine | 1.6869 | 0.0452 | 1.6683 |
| N-acetyl-d-glucosamine 6-phosphate | -0.6962 | 0.0458 | 1.6472 |
| Asn-Trp | -0.2663 | 0.0461 | 1.6662 |
| Isopropyl 4-hydroxybenzoate | -0.1503 | 0.0470 | 1.6366 |
| sn-Glycerol 3-phosphoethanolamine | -0.4022 | 0.0473 | 1.6224 |
| Capric acid | -0.1385 | 0.0476 | 1.6406 |
| 1-Palmitoyl-2-arachidonoyl-sn-glycero-3-phosphocholine | -0.3993 | 0.0482 | 1.6394 |
| Tremulacin | -1.3221 | 0.0483 | 1.5925 |
| Pc(16:0e/8-hepe) | -0.3217 | 0.0491 | 1.6393 |

log2(FC), log2(Fold Change); VIP-value, value of variable importance in projection.
