# Supplementary material for: Untargeted Metabolomics Reveals the Effect of Selective Breeding on the Quality of Chicken Meat
Source: Metabolites. 2022 Apr 19;12(5):367. doi: 10.3390/metabo12050367 (PMC9144515; doi:10.3390/metabo12050367)
Supplement: Supplementary file 1 [file metabolites-12-00367-s001.zip › Table S2. Differentiated metabolites in thigh muscle between line S and line D.pdf]

Table S2. Differentiated metabolites in thigh muscle between line S and line D.

Metabolite name	log2(FC)	p-value	VIP-value
Uridine 5'-diphosphoglucuronic acid (UDP-D-glucuronate)	-0.9426	0.0002	2.3177
2-Linoleoylglycerol	1.2166	0.0016	2.2838
1,2-Di-(13z-docosenoyl)-sn-glycero-3-phosphocholine	-0.9746	0.0016	2.0315
Flavin adenine dinucleotide (FAD)	7.4928	0.0022	2.1318
Ranaconitine	7.0427	0.0022	2.1813
Trifluperidol	7.2222	0.0023	1.9877
Cytidine monophosphate n-acetylneuraminic acid	-0.4324	0.0032	1.9736
2-Amino-2-methyl-1,3-propanediol	1.1294	0.0041	2.2963
Choline	-1.0053	0.0055	1.9597
Creatinine	0.9454	0.0057	2.2421
3,4-Dihydro-6-hydroxy-.alpha.,2,5,7,8-pentamethyl-2h-1-benzopyran-2-pentanoic acid	0.535	0.0059	2.2875
D-proline	0.6492	0.0062	2.1134
N-acetyl-L-alanine	-1.1098	0.0064	1.4008
Ser-His	-0.8336	0.0067	2.1372
Octyl-3,5-di-tert-butyl-4-hydroxyhydrocinnamate	1.5025	0.0068	2.21
Fructose	0.505	0.0069	2.2644
Dethiobiotin	-0.5875	0.0073	1.8179
Caffeine	1.2194	0.0078	2.0762
L-palmitoylcarnitine	-1.6521	0.0082	2.1876
Thr-Gly-Thr	1.3362	0.0084	2.2977
Glycerophosphocholine	-1.3493	0.0087	2.0717
Uridine 5'-triphosphate	1.6568	0.0091	2.1886
Schisandrol a	-1.0999	0.0095	1.796
Medicarpin	-1.135	0.0102	1.9124
Arg-Asn	1.249	0.0105	2.1726
Myristoyl-l-carnitine	-1.189	0.0105	1.7818
1-Methyl-l-histidine	0.3534	0.0108	2.229
Trigonelline	1.1452	0.0113	2.1258
5-Ethyl-2'-deoxyuridine	0.786	0.0116	2.1386
Benzoic acid, 3-[(e)-[(3ar,4s,5s,6ar)-4-[(1e,3r)-3-cyclohexyl-3-hydroxy-1-propen-1-yl]hexahydro-5-hydroxy-2(1h)-pentalenylidene]methyl]-, rel-	1.1789	0.0117	2.1323
L-myo-inositol-1,4,5-triphosphate	2.5755	0.0119	2.0995
DL-serine	1.1069	0.0125	2.0579
Dioctyl phthalate	-0.5059	0.0134	1.5961
DL-asparagine	-0.3414	0.0136	1.4911
Oleoyl-l-carnitine	-1.4032	0.0138	1.8713
Schizandrin	0.7874	0.0141	2.1441

4-Pregnen-6.beta.,11.beta.,17,21-tetrol-3,20-dione	0.9879	0.0146	2.001
L-glutamic acid, l-methionyl-l-alanylglycyl-l-prolyl-l-histidyl-l-prolyl-l-valyl-l-isoleucyl-l-valyl-l-isoleucyl-l-threonylglycyl-l-prolyl-l-histidyl-l-.alpha.-glutamyl-	-7.0057	0.0148	1.7598
Proscillaridin a	-5.3716	0.0151	1.5325
His-Ile	6.5414	0.0152	1.8716
Phe-Arg	0.9447	0.0153	1.9193
Enoxacin	-0.8553	0.0164	1.5345
Ergocristine	4.5398	0.0166	1.7578
Diethanolamine	0.5221	0.017	2.1023
Isopentenyl pyrophosphate	2.3066	0.0175	2.1435
D-mannose 6-phosphate	3.3221	0.0176	2.1003
Madecassic acid	1.0749	0.0181	2.0967
3'-O-methylcytidine	0.7753	0.0187	1.7144
M-Chlorohippuric acid	1.0912	0.0188	1.6569
L-hydroxyarginine	0.5345	0.0191	2.0553
D-glucosamine 6-phosphate	1.8676	0.0193	2.0222
D-mannose-6-phosphate	3.4786	0.0197	1.9652
Ile-Lys	2.1177	0.0202	2.0892
Alpha-D-galactose 1-phosphate	2.5917	0.0205	1.8555
Uridine 5'-diphosphate	-1.0995	0.0217	1.7173
Curcumin	1.2996	0.0238	1.4578
6-Phosphogluconic acid	2.2615	0.0241	2.1674
D-erythrose 4-phosphate	2.8889	0.0243	2.1293
10,12-Tricosadiynoic acid	-2.0226	0.0251	2.0217
1-O-hexadecyl-2-deoxy-2-thio-s-acetyl-sn-glyceryl-3-phosphorylcholine	-0.6482	0.026	1.3366
1-(1',3'-Benzodioxol-5'-yl)-2-butanamine	-0.8277	0.0262	1.4777
Cholesteryl sulfate	-0.9879	0.0266	1.3521
Tri(3-chloropropyl) phosphate	1.4469	0.0267	1.9463
Norethindrone	-0.8971	0.0267	1.6427
Asp-Arg	1.0631	0.0268	1.9752
Guanosine	0.7259	0.0269	2.0322
2-Keto-3-deoxyoctonic acid	1.1191	0.0271	1.8573
5-Iodo-2'-deoxycytidine	3.2485	0.0273	2.2128
3,4-Dichlorophenol	-0.4165	0.0274	1.2151
Alpha-D-glucose	1.4141	0.0274	1.899
D-myo-inositol-1,3-diphosphate	2.3505	0.0275	2.0489
D-allose	1.2201	0.0276	1.5429
Ile-Asp	1.0922	0.0277	1.8624
Ile-Asn	1.1975	0.028	1.693
Phosphocholine	-1.2207	0.0281	1.7439
Demethoxycurcumin	0.846	0.0284	1.7967

1,2-Diamino-2-methylpropane	-0.4241	0.0305	1.4901
N-methyl-l-threonine	0.5014	0.0314	1.8687
UDP-D-galactose	-1.0698	0.0326	1.591
N-acetylmannosamine	-0.9923	0.0329	1.6835
1-Palmitoyl-2-docosahexaenoyl-sn-glycero-3-phosphocholine	0.4411	0.033	1.931
7-Chloro-3-methylquinoline-8-carboxylic acid	0.7063	0.0333	1.8956
Thr-Ala	1.6183	0.0343	1.6395
UDP-D-glucose	-1.0225	0.0343	1.6468
Arg-Asp	0.5445	0.0344	1.8711
Gly-Leu-Arg	-1.4654	0.0347	1.6575
D-ribulose 5-phosphate	0.8472	0.035	1.9468
1h-Indazole-3-carboxamide, n-[(1s)-1-(aminocarbonyl)-2-methylpropyl]-1-(cyclohexylmethyl)-	-0.6823	0.0361	1.4646
Val-Asp	1.4202	0.0366	1.668
Loureirin a	0.991	0.0372	1.6026
1,2-Di-(9z,12z,15z-octadecatrienoyl)-sn-glycero-3-phosphocholine	0.7319	0.0373	1.8708
4,2'-Dihydroxy-3,4',6'-trimethoxychalcone	0.6765	0.0375	1.7865
4,4'-Methylenebis(2,6-di-tert-butylphenol)	-0.7588	0.0376	1.3865
Arg-Ala	2.0195	0.0378	1.5815
Gentiopicroside	-1.5248	0.0392	1.6891
1-Stearoyl-2-arachidonoyl-sn-glycero-3-phospho-(1'-myo-inositol)	-0.6963	0.0401	1.4127
D-(+)-mannose	1.2342	0.0404	1.4505
L-gulono-1,4-lactone	0.9471	0.0406	1.6704
DL-2-aminoadipic acid	0.8743	0.0406	1.7897
1h-Imidazo[4,5-c]pyridine-6-carboxylic acid, 1-[[4-(dimethylamino)-3-methylphenyl]methyl]-5-(2,2-diphenylacetyl)-4,5,6,7-tetrahydro-, (6s)-	-1.0116	0.0407	1.48
Nicotinuric acid	1.5501	0.0409	1.0774
D-mannose 1-phosphate	1.7568	0.0413	1.6319
Adenosine 3'-monophosphate	0.3885	0.0422	1.8625
Ginsenoside f1	-0.5372	0.0423	1.2331
Dl-lactate	0.5307	0.0429	1.8316
Matairesinol	1.2242	0.0444	1.3313
Glyceric acid	0.1486	0.045	2.106
2'-O-methylinosine	0.4203	0.0452	1.9272
2-Hydroxy-6-methylquinoline-3-carbaldehyde	0.2534	0.0453	2.0406
Leu-Ser	1.704	0.0458	1.4749
D-tagatose	1.047	0.0464	1.3456
Clomazon	0.6995	0.0464	1.8696
D-fructose-6-phosphate	2.1535	0.0467	1.5932

2'-Deoxyguanosine 5'-monophosphate (dGMP)	-0.5298	0.0467	1.2137
Val-Asn	1.0531	0.0496	1.4909

log2(FC), log2(Fold Change); VIP-value, value of variable importance in projection.
