# Supplementary figures and images for: Untargeted Metabolomics Reveals the Effect of Selective Breeding on the Quality of Chicken Meat
Source: Metabolites. 2022 Apr 19;12(5):367. doi: 10.3390/metabo12050367 (PMC9144515; doi:10.3390/metabo12050367)

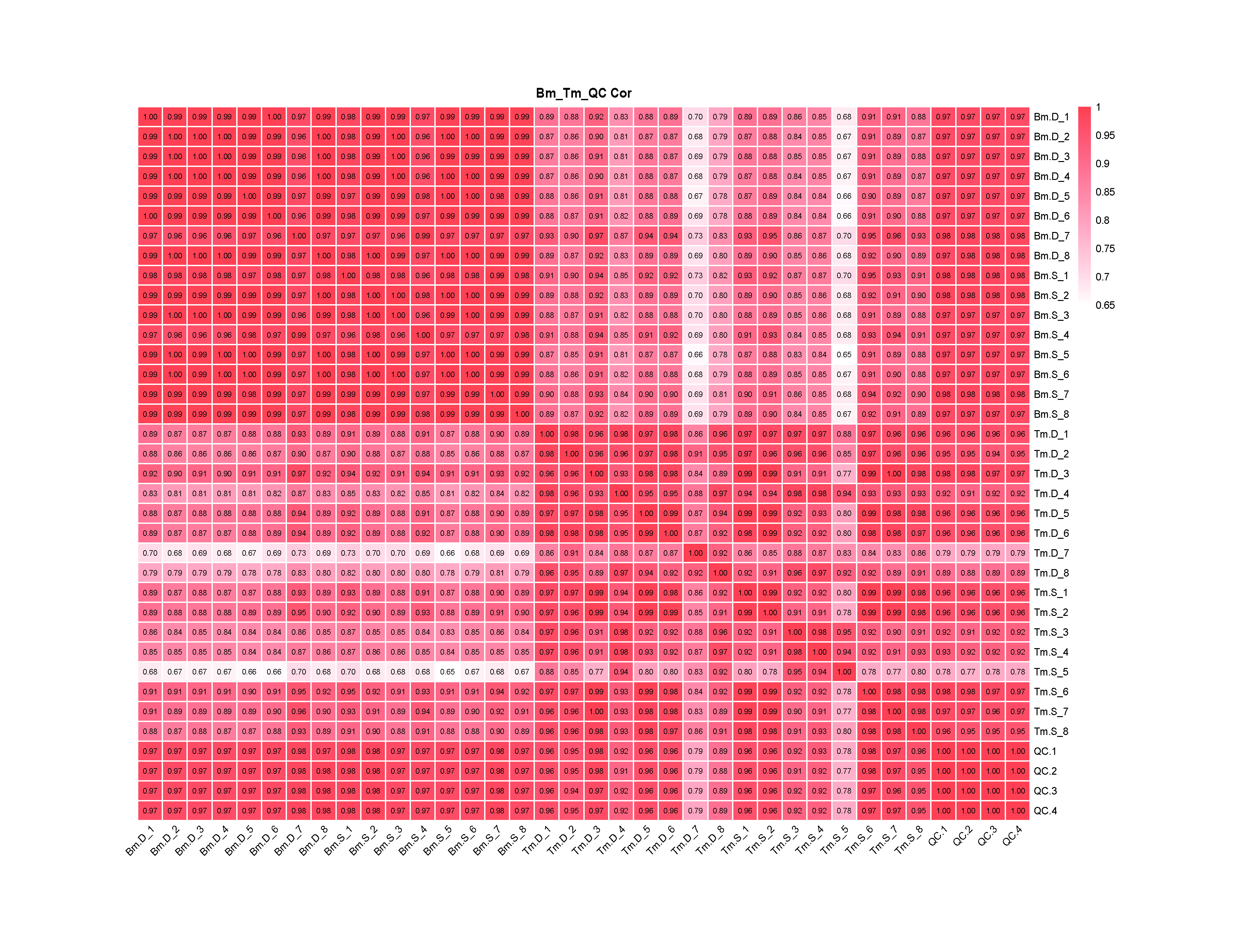

Supplement: Supplementary file 1 [file metabolites-12-00367-s001.zip › Figure S1. The heatmap of correlations between all samples.tif]

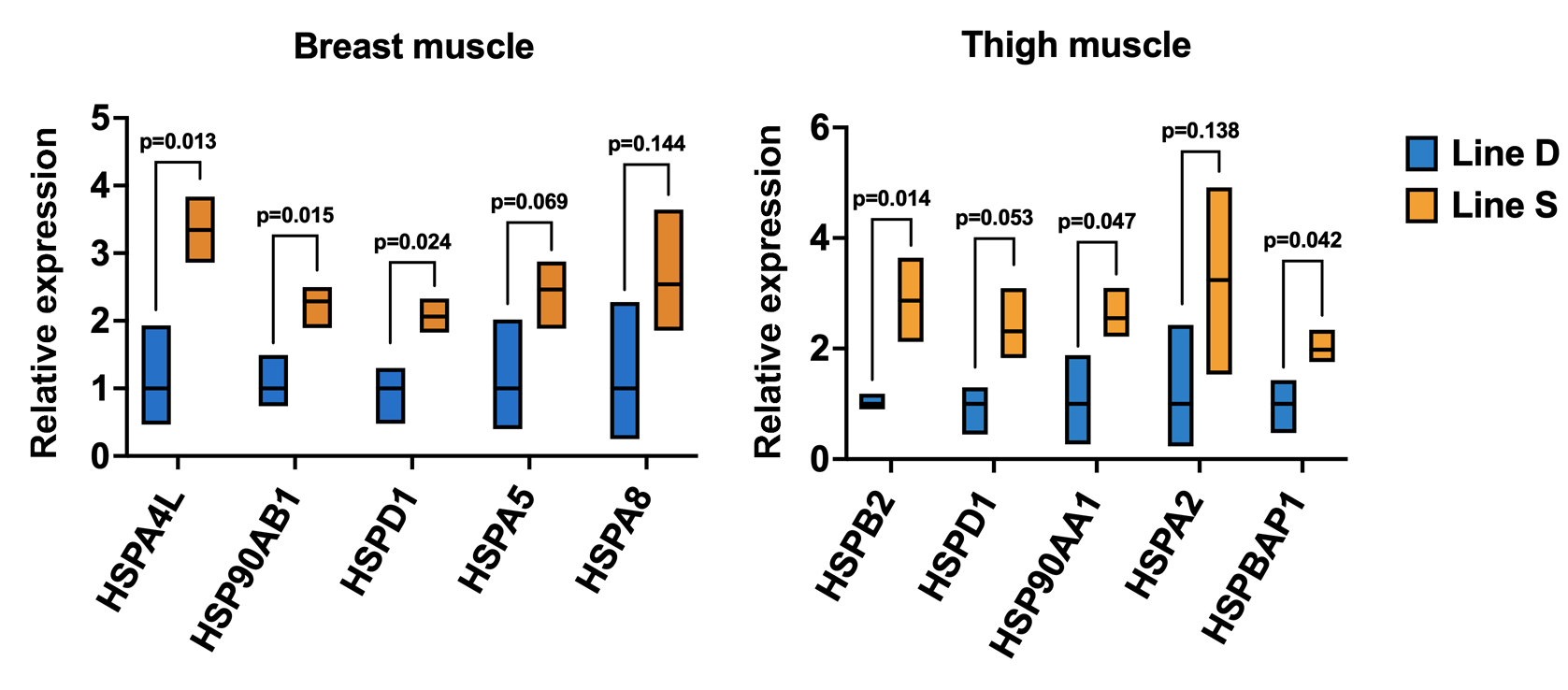

Supplement: Supplementary file 1 [file metabolites-12-00367-s001.zip › Figure S2. The relative expression of several genes from RNA-seq.tif]
